# Dye coupling of horizontal cells in the primate retina

**DOI:** 10.3389/fopht.2023.1173706

**Published:** 2023-11-16

**Authors:** Feng Pan, Stephen C. Massey

**Affiliations:** ^1^ School of Optometry, The Hong Kong Polytechnic University, Hong Kong, Hong Kong SAR, China; ^2^ Centre for Eye and Vision Research (CEVR), Hong Kong, Hong Kong SAR, China; ^3^ Research Centre for SHARP Vision (RCSV), The Hong Kong Polytechnic University, Hong Kong, Hong Kong SAR, China; ^4^ Ruiz Department of Ophthalmology and Visual Science, McGovern Medical School, University of Texas at Houston, Houston, TX, United States

**Keywords:** horizontal cells, retina, gap junction, glutamate receptors, photoreceptors

## Abstract

In the monkey retina, there are two distinct types of axon-bearing horizontal cells, known as H1 and H2 horizontal cells (HCs). In this study, cell bodies were prelabled using 4′,6-diamidino-2-phenylindole (DAPI), and both H1 and H2 horizontal cells were filled with Neurobiotin™ to reveal their coupling, cellular details, and photoreceptor contacts. The confocal analysis of H1 and H2 HCs was used to assess the colocalization of terminal dendrites with glutamate receptors at cone pedicles. After filling H1 somas, a large coupled mosaic of H1 cells was labeled. The dendritic terminals of H1 cells contacted red/green cone pedicles, with the occasional sparse contact with blue cone pedicles observed. The H2 cells were also dye-coupled. They had larger dendritic fields and lower densities. The dendritic terminals of H2 cells preferentially contacted blue cone pedicles, but additional contacts with nearly all cones within the dendritic field were still observed. The red/green cones constitute 99% of the input to H1 HCs, whereas H2 HCs receive a more balanced input, which is composed of 58% red/green cones and 42% blue cones. These observations confirm those made in earlier studies on primate horizontal cells by Dacey and Goodchild in 1996. Both H1 and H2 HCs were axon-bearing. H1 axon terminals (H1 ATs) were independently coupled and contacted rod spherules exclusively. In contrast, the H2 axon terminals contacted cones, with some preference for blue cone pedicles, as reported by Chan and Grünert in 1998. The primate retina contains three independently coupled HC networks in the outer plexiform layer (OPL), identified as H1 and H2 somatic dendrites, and H1 ATs. At each cone pedicle, the colocalization of both H1 and H2 dendritic tips with GluA4 subunits close to the cone synaptic ribbons indicates that glutamate signaling from the cones to H1 and H2 horizontal cells is mediated by α-amino-3-hydroxy-5-methyl-4-isoxazolepropionic acid (AMPA) receptors.

## Introduction

Horizontal cells (HCs) are located in the distal inner nuclear layer of the retina and provide feedback to photoreceptors in the outer plexiform layer (OPL) (1–5). The inhibition of photoreceptors generates a signal common to all downstream neurons and is believed to contribute to contrast enhancement, color opponency, and the generation of center-surround receptive fields in cones, and to a higher level of functional specificity of the retinal ganglion cell output (3, 4, 6–8). The inhibitory feedback signal from HCs to photoreceptors may be transmitted by the modulation of calcium channels, hemichannels, and gamma*-*aminobutyric acid (GABA) release. However, the exact details of this feedback loop remain controversial (1, 4, 9–12).

Most mammalian species have two types of HCs although the mouse retina is a notable exception, with only one HC type (13). In the primate retina, there are two HC types, both axon-bearing, known as H1 and H2 (14, 15). The somatic dendrites of H1 HCs contact all red/green cone pedicles but have sparse or no contact with blue cones. H1 axons expand into elaborate axon terminals (ATs), which contact rod spherules exclusively. In contrast, the dendrites of H2 HCs invaginate most cone pedicles but have many contacts with blue cones and sparse contact with red/green cones. The H2 axons have few axon collaterals, which contact only cones.

The original descriptions of primate HCs were taken from Golgi and electron microscope studies. More recently, primate HCs were stained with the lipophilic dye Dil or by filling single cells with Neurobiotin, a neuronal tracer (15–17). The H1 and H2 HCs are both independently coupled and, after dye injection, Neurobiotin spreads via gap junctions to adjacent cells of the same type, revealing a patch of coupled cells. The axons can be observed leaving such patches of coupled H1 or H2 HCs, but, due to the length of the axon, the staining fades before the details of the AT are revealed. However, by blocking gap junctions, to reduce the spread of Neurobiotin through the coupled network, the morphology of the ATs can be examined. In this study, we used Neurobiotin injections of HCs to reveal the details of photoreceptor contacts, and, in particular, the cone contacts made by H1 and H2 dendrites.

The classic postsynaptic structure below each cone synaptic ribbon is known as a triad and it consists of two flanking HC dendrites and a central bipolar cell process (18). These invaginating bipolar dendrites are predominantly ON cone bipolar cells, whereas some OFF cone bipolar cells make flat contacts along the base of the cone pedicle, which is somewhat distant from the synaptic ribbon. The cones communicate with HCs and bipolar cells using glutamate as a neurotransmitter (19), and the distribution of glutamate receptors at cone pedicles has been described in detail (20, 21).

There are three main groups of postsynaptic ionotropic glutamate receptors based on their response to glutamate agonists. The groups are named according to the updated glutamate receptor nomenclature (22): (1) α-amino-3-hydroxy-5-methyl-4-isoxazolepropionic acid (AMPA) receptors, including the GluA1, GluA2, GluA3, and GluA4 subunits. AMPA receptors mediate rapid synaptic transmission in the central nervous system. (2) Kainate (KA) receptors include GluK1, GluK2, GluK3, GluK4 (KA1), and GluK5 (KA2). Kainate receptors also play a role in synaptic transmission in the central nervous system and carry signals between cones and certain OFF bipolar cells. (3) *N*-methyl-D-aspartate (NMDA) receptors (NR1, NR2A, NR2B, NR2C, and NR2D) (23), which do not participate in the OPL. In addition, eight metabotropic GluRs (i.e., mGluR1–mGluR8) have been identified in vertebrates, which can be subdivided into three groups based on differences in amino acid sequence, signal transduction mechanism, and pharmacological profiles (24–27). mGluR6, also known as GRM6, is expressed only by ON cone bipolar cells and rod bipolar cells in the retina (28).

The AMPA receptors containing the GluA4 subunits are found in two bands (the upper and lower bands) under each cone pedicle. The upper band is very close to, and aligned with, the cone synaptic ribbons, whereas the lower band is found at desmosome-like junctions among HC dendrites, distinctly below the cone pedicle (20, 21). Electron microscopy (EM) showed that the GluA4 subunits occur at each triad, in which the two HC dendrites abut just below the cone synaptic ribbon (21). Although this position is consistent with the expression of GluA4 by HCs, it was not possible to identify which processes were derived from H1 or H2 HCs.

In this article, we combined the injection of H1 and H2 HCs with Neurobiotin with immunolabeling and confocal microscopy to demonstrate that the dendritic tips of both H1 and H2 HCs express GluA4 subunits at a site that is close to, and aligned with, the cone synaptic ribbons. This indicates that cones drive HC responses via AMPA receptors.

## Methods

### Preparation of isolated retina

For this study, seven pieces of retina from four macaques were used. The pieces of the peripheral retina from an adult macaque (*Macaca mulatta*; kindly provided by Dr. David W. Marshak, Department of Neurobiology & Anatomy, University of Texas Medical School at Houston and Dr. Samuel M. Wu, Cullen Eye Institute, Baylor College of Medicine at Houston) were immersed in oxygenated (95% O_2_/5% CO_2_) Ames’ medium. The retinal cells were then prelabled by incubating in Ames’ medium with 5 μM 4,6-diamino-2-phenylindole (DAPI) for 30 min. There were approximately 30 synaptic ribbons per cone; this is consistent with a midperipheral location (18, 21).

### Injection of Neurobiotin

The DAPI-labeled cells were visualized using a fixed-stage Olympus BX-50WI epifluorescent microscope (Tokyo, Japan). The cells were impaled under visual control using pipette tips filled with 4% Neurobiotin (Vector Laboratories, Burlingame, CA, USA) and 0.5% Lucifer Yellow CH (Molecular Probes, Eugene, OR, USA) in double-distilled water, and were then backfilled with 3 M LiCl. Alexa Fluor 568™ (Molecular Probes, A10437)-treated cells were backfilled with 3 M KCl. The electrode resistance was ≈ 100 MΩ. The impaled cells were then injected with a biphasic current (+1.0 nA, 3 Hz) for 10 min. After the last injection was administered, the retinal pieces were fixed in 4% paraformaldehyde for 30 min, then washed six times in phosphate buffer (PB) and blocked in 3% donkey serum in PB before further immunocytochemical experiments were carried out. Meclofenamic acid (MFA) (Sigma, M-4531) at a concentration of 100 µM was used to block gap junctions (29).

### Immunocytochemistry

After fixation, the tissues were washed extensively with 0.1 M PB (pH 7.4) and blocked with 3% donkey serum in 0.1 M PB with 0.5% Triton-X 100 and 0.1% NaN_3_ overnight. The antibodies were diluted in 0.1 M PB with 0.5% Triton-X 100 and 0.1% NaN_3_ containing 1% donkey serum. The tissues were then incubated in primary antibodies for 3–7 days at 4°C and, after extensive washing, incubated in secondary antibodies overnight at 4°C. After washing with 0.1 M PB, the tissues were mounted in VECTASHIELD^®^ (Vector Laboratories) for observation.

The secondary antibodies used were donkey anti-rabbit or anti-goat Cy3^®^ (1: 200) and donkey anti-mouse or anti-goat Cy5^®^ (1: 200) (Jackson ImmunoResearch Laboratories, West Grove, PA, USA). Neurobiotin was visualized with Alexa Fluor 488™-conjugated streptavidin (Molecular Probes) or Cy3-conjugated streptavidin (Jackson ImmunoResearch Laboratories, West Grove, PA, USA).

A rabbit polyclonal antibody against GRM6 (mGluR6) in primate retina (1: 1,000), kindly provided by Dr. Noga Vardi [Research Resource Identifier (RRID): AB 2314792], stained the same punctate structures in the OPL, as previously reported, in a manner that was consistent with the distribution of GRM6 receptors (30). An affinity-purified polyclonal rabbit antibody against the C-terminus (RQSSGLAVIASDLP) of rat glutamate receptor subunit 4 (GluA4) was purchased from Chemicon International (Millipore catalog number AB1508; RRID: AB 90711; 1: 200). A Western blot, conducted in accordance with the manufacturer’s technical instructions. These antibodies have been shown to label glutamatergic synapses in rabbit retinas (30–32).

GluK1(GluR5) (C-18; SC-7616; RRID: AB 641048) and GluK1 (N-19; SC-7617; RRID: AB 641050) are affinity-purified goat polyclonal antibodies that were raised against the synthetic peptides comprising the amino acid sequence 900–918 at the C-terminus and the amino acid sequence 1–20 at the N-terminus of GluR-5 of human origin (Santa Cruz Biotechnology, Santa Cruz, CA, USA; 1: 100). Both antibodies stained the same pattern of receptors in the OPL.

A mouse-purified monoclonal antibody against a recombinant protein consisting of the amino acid sequence 361–445 of the C-terminal-binding protein 2 (Ctbp2), which is a RIBEYE homolog, was purchased from BD Biosciences (San Diego, CA, USA; number 612044; RRID: AB 399431; 1: 500). The antibody was generated against mouse Ctbp2, and it has been shown to recognize synaptic ribbons in mammalian retinas (33, 34). The staining patterns for the Ctbp2 antibody in the mammalian retina are well known.

### Confocal microscopy

Images were acquired on a Zeiss LSM-510 (Zeiss, Thornwood, NY) confocal microscope using a ×63 objective (N.A. 1.4). The alignment for all three channels and resolution were checked at ×8 zoom using 1-μm fluorescent spheres (molecular probes). The XY resolution of the instrument was 200 nm–300 nm, and all three channels were superimposed. The *z*-axis steps were usually 0.4 μm, and the resulting images are presented as short stacks of 4–6 optical sections (2 μm–3 μm) to compensate for the slight ripples across the tissue and present an even plane of focus. The *z*-axis reconstructions were oversampled in 0.2-μm or 0.3-μm steps and reconstructed in Zeiss software. For image stacks, the brightest value in the *z*-dimension was presented for any *xy* pixel. This had the effect of flattening the image. The brightness, contrast, and color balance of the digital images were adjusted in Adobe PhotoShop (Adobe Systems, San Jose, CA, USA), but no filtering or region-specific adjustments were made to any of the images.

Statistical analyses were carried out using Origin software (OriginLab, Northampton, MA, USA). The statistical significance (*p* < 0.05) was determined using a Student’s *t*-test. The results shown are mean values ± standard errors of the mean (SEMs) unless otherwise indicated.

## Results

### The mosaic of rods and cones was marked by glutamate receptors

**Figure 1:**
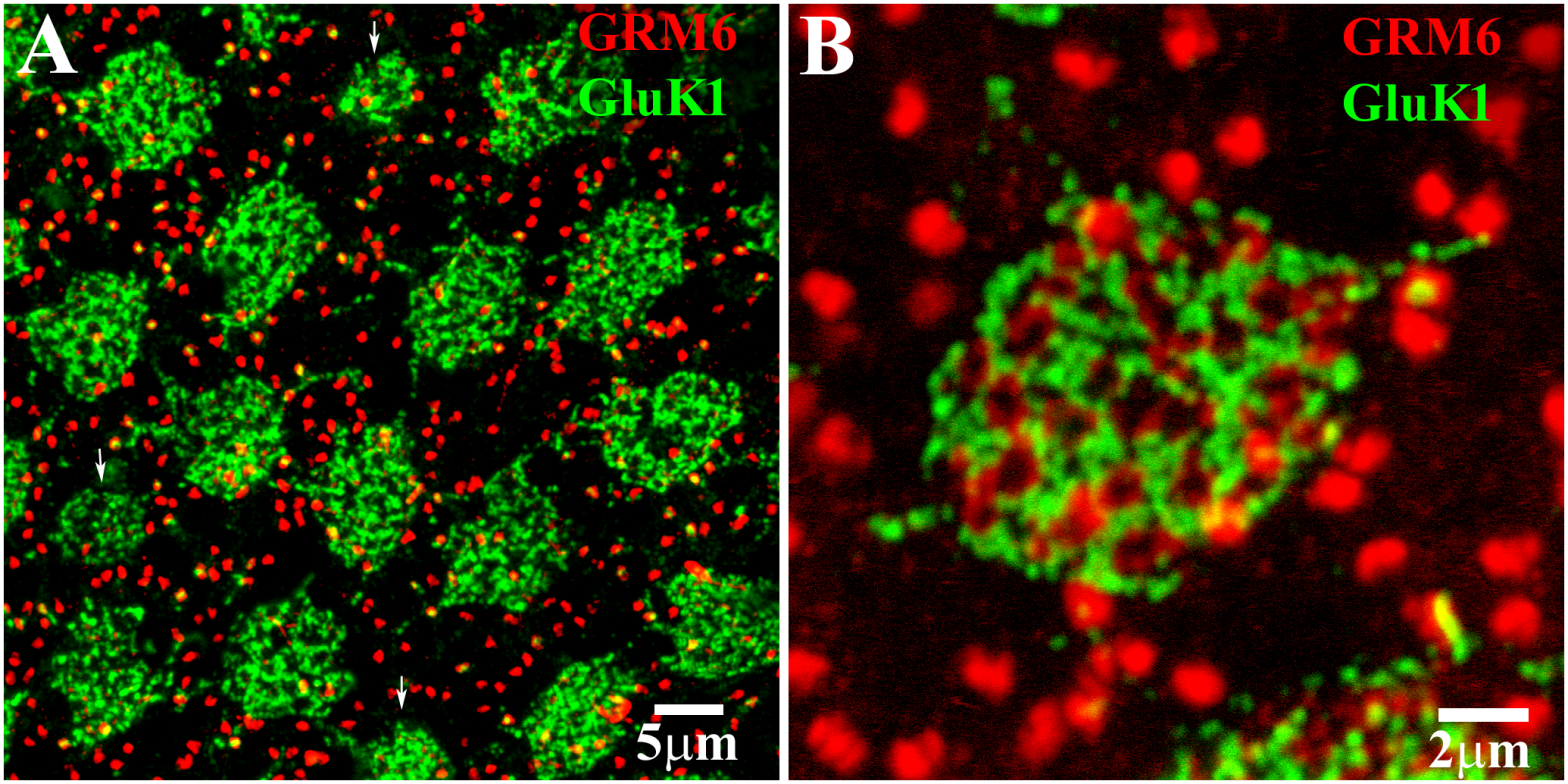
Use of GRM6 and GluK1 to map the positions of cones and rods. **(A)** GRM6-labeled ON bipolar dendrites and GluK1-labeled OFF cone bipolar dendrites were used to map the position of cone pedicles. The arrows show the position of the blue cone pedicles, which were smaller than the red/green cones. The bright-red doublets of GRM6 labeling of rod bipolar dendrite tips indicate the position of the rod spherules. Scale bar = 5 μm. **(B)** Double-label image at a single cone pedicle marked by a cluster of fine GRM6-labeled ON bipolar dendrites, and GluK1-labeled OFF cone bipolar dendrites. The rod spherule positions are indicated by the bright-red GRM6 doublets (usually) of the rod bipolar dendrites. Scale bar = 2 μm.

The glutamate receptors GluK1 and GRM6 are important to visual signaling in the retina. GluK1 is a type of kainate receptor expressed by the dendrites of certain OFF cone bipolar cells at each cone pedicle (35–41). On the other hand, GRM6 (a metabotropic glutamate receptor) exhibits two different profiles in the retina (30). Antibodies against GRM6 label the dendritic tips of rod bipolar cells at each rod spherule, which results in the presence of two bright spots in close proximity to each other. GRM6 also showed clusters of fine terminals at the position of each cone pedicle, which showed the position of ON cone bipolar cell dendrites. Antibodies that target these receptors can be used to label and map the position of cone pedicles (clusters of GluK1 and GRM6 labeling) and rod spherules (GRM6 doublets) in the retina (**Figure 1A**) (30, 37). By using the double labeling of GluK1 and GRM6 in the whole-mount retina, it is possible to visualize the photoreceptor contacts of the different HC types in the retina. This technique can be used to accurately determine the position of the cone pedicles or rod spherules (GRM6 doublets) in the retina (**Figure 1B**). Although this example uses GluK1 and GRM6, similar results can be obtained using GluA4 to mark cone pedicles (see below) (37).

The size of red and green cone pedicles in the midperiphery of the macaque retina was 58.7 µm^2^ ± 1.3 µm^2^ (mean ± SEM; *n* = 71), whereas the size of blue cone pedicles was approximately half that of the red/green cone pedicles at around 31.1 µm^2^ ± 1.1 µm^2^ (mean ± SEM; *n* = 13). The blue cone pedicles also protrude a little further into the OPL (42). These distinctions allow the differentiation of red and green cones from blue cones.

### H1 horizontal cells

**Figure 2:**
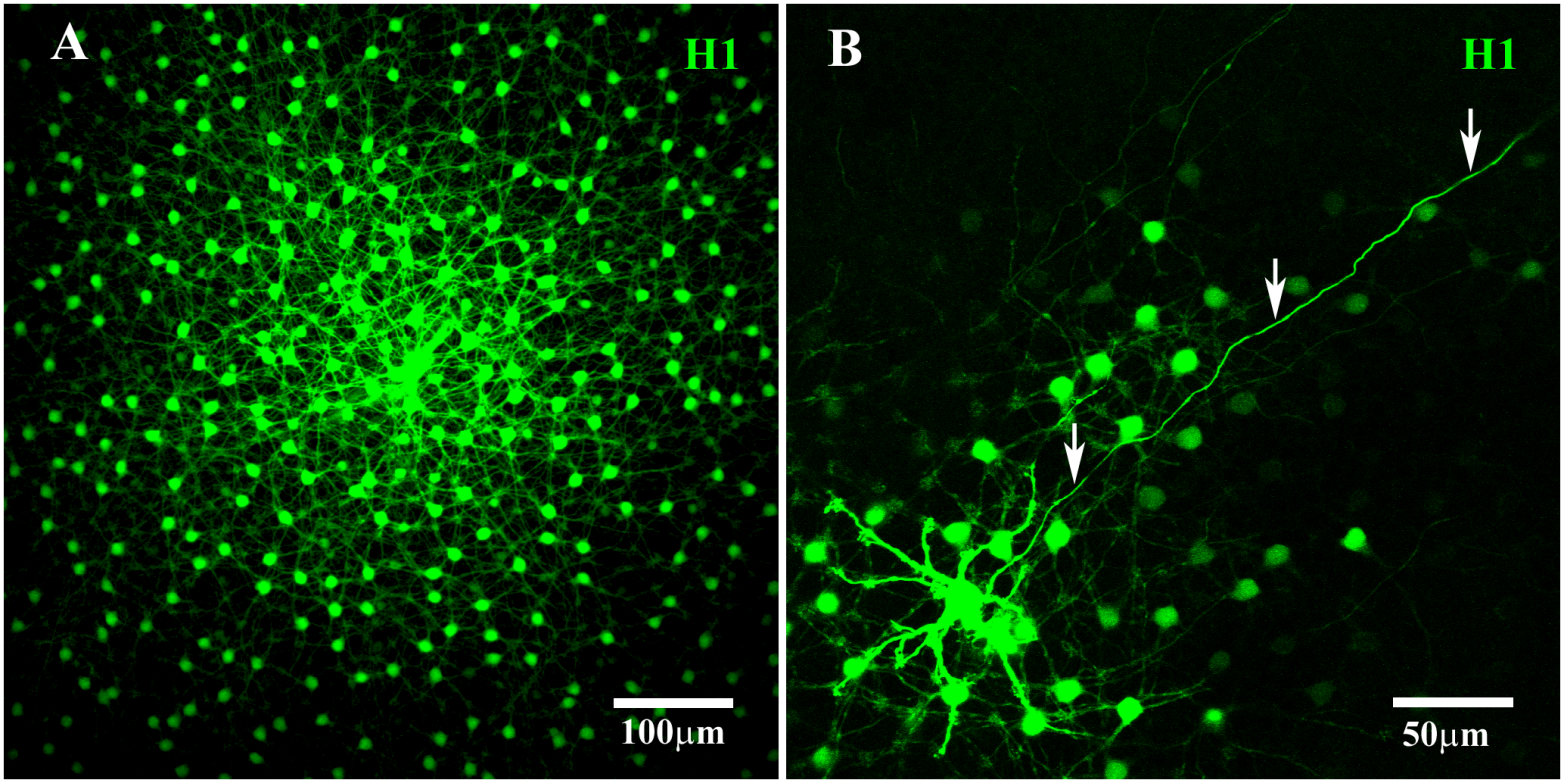
H1 coupling in the primate retina. **(A)** A prominent mosaic of dye-coupled H1 HCs was labeled together with a dense matrix of overlapping dendrites with many fine terminal clusters after Neurobiotin injection into a single H1 soma. Scale bar = 100 μm. **(B)** Neurobiotin-filled axonal terminals (arrows) were visualized after blocking gap junctions with 100 μM meclofenamic acid to reduce the spread of Neurobiotin through the coupled network. This axon ran for more than 400 μm before fading out. Scale bar= 50 μm. H1 horizontal cells (H1 HCs).

**Figure 3:**
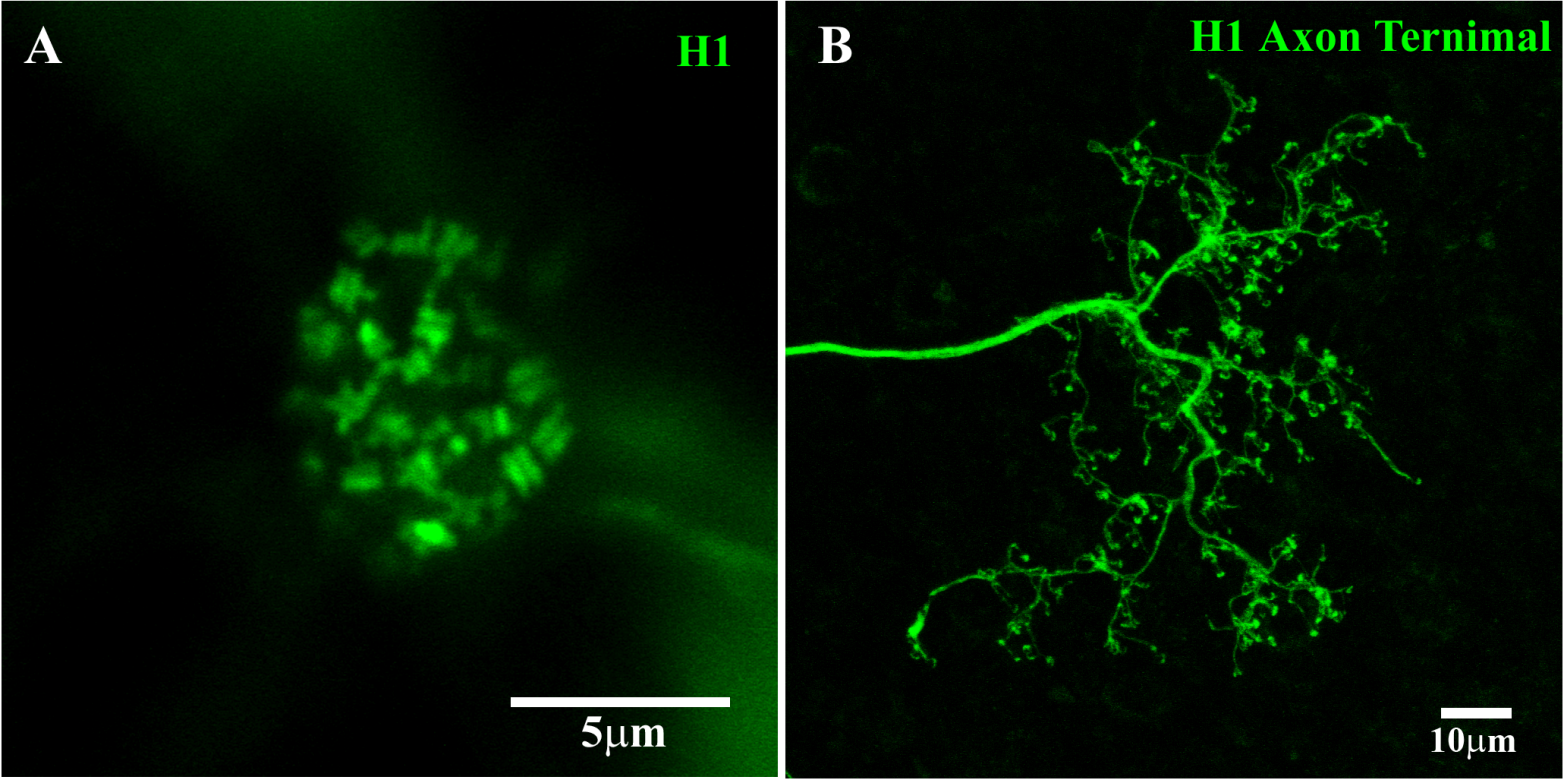
H1 dendritic terminals and axon terminal. **(A)** H1 dendrites at a single cone pedicle within a field of dye-coupled H1 HCs. The H1 dendrites from multiple horizontal cells in the network converged at each cone pedicle, with two terminals forming an equals sign at each cone synaptic ribbon. The number of H1 dendrites also identified this cone as a red/green cone. This figure shows a Neurobiotin fill, visualized with streptavidin conjugated with Alexa Fluor 488. Scale bar = 5 μm. H1 horizontal cell (H1 HC). **(B)** A single H1 was dye-injected with Neurobiotin and visualized using Alexa Fluor 488 and gap junctions were blocked with 200 μM MFA. Under these conditions, Neurobiotin passed down the axon to fill the elaborate AT structure. There were many fine endings, which were horseshoe or question mark shaped (detailed images shown in **Figure 4**). The H1 ATs made contact with the rod spherules. This figure shows a Neurobiotin fill, visualized with streptavidin conjugated with Alexa Fluor 488. Scale bar = 10 μm.

The H1 HCs in the primate retina were found to be axon-bearing, similar to B-type HCs in the rabbit retina. In DAPI-labeled retinas, the somas of the H1 HCs were large (72 µm^2^ ± 9.7 µm^2^, average ± SEM; *n* = 78), bright, and round. After Neurobiotin injection into a single soma, an extensive matrix of H1 coupling was revealed, with somatic dendrites and their fine terminals spread out across more than 500 µm (**Figure 2A**). Furthermore, the somatic dendrites of the H1 HCs converged to form dense clusters at most cone pedicles (**Figure 3A**). Those cone pedicles with few or no H1 dendrites were smaller and thus identified as blue cone pedicles (see below). Therefore, the H1 HCs preferentially contacted red/green cones, as previously reported (15, 16).

After filling the H1 population, the dense clusters of terminal dendrites at the red/green cone pedicles indicate contributions from multiple H1 horizontal cells. This is consistent with the coverage factor of approximately three to four for H1 HCs (43, 44). With the focus on the cone pedicle base, the H1 dendrites formed a pattern of equals sign (=) extending across most synaptic ribbons, suggesting the presence of two laterally placed H1 HC dendrites at each synaptic ribbon (**Figure 3A**) (18). In previous studies, when a single H1 HC was labeled, only one lateral position at each cone synaptic ribbon was stained (17, 20, 45). This indicates that the H1 HC processes at a single ribbon usually come from two different HCs.

In one well-stained example, we counted the terminal pairs or clusters of H1 HC dendrites at 20 red/green cone pedicles. There were 27 ± 0.6 (mean, ± SEM; *n* = 20) H1 dendritic pairs or clusters per cone pedicle, close to the number of synaptic ribbons. This falls within the range of 20–50 synaptic ribbons per cone pedicle previously reported (18, 21) and it is consistent with a mid-peripheral location of the retinal sample (46). If it is presumed that there are two H1 processes per ribbon, this suggests there are more than 50 H1 dendrites per cone terminal, at this retinal eccentricity. However, this may be a slight overestimate because we have not accounted for the innervation from H2 HCs (see below).

### H1 HC axon terminal

Neurobiotin injection provided only poorly filled AT structures due to its spread through the somatic network, and the high resistance of the axon. The staining of primate H1 ATs was improved through the application of the gap junction antagonist MFA (29) before the Neurobiotin injection. This reduced or prevented the spread of Neurobiotin through the H1 network and accentuated the axon staining. Perfusion with 100 μM MFA blocked much of the H1 coupling, revealing a well-stained axon (**Figure 2B**). However, despite following the axon for a few hundred microns before the staining faded, the AT was not visible. This is consistent with previous reports in which the axons of H1 HCs were reported to be 1.5 mm–2.5 mm in length (43). In comparison, B-type ATs in the rabbit retina were stained completely by filling the somas in the presence of 100 μM MFA, but the axons were shorter (approximately 500 μm in length), facilitating the diffusion of Neurobiotin to the terminal (47).

By increasing the concentration of MFA to 200 μM and injecting Neurobiotin into a single H1 AT, the whole AT structure was visualized in great detail, as shown in **Figure 3B**. This method retained Neurobiotin inside the injected cell, allowing it to pass through the axon and fill the intricate AT structure. The H1 HC can be divided into three distinct parts: the soma, axon, and AT. The AT endings take the form of horseshoe- or question mark-shaped structures and make contact with the rod spherules (48).

**Figure 4:**
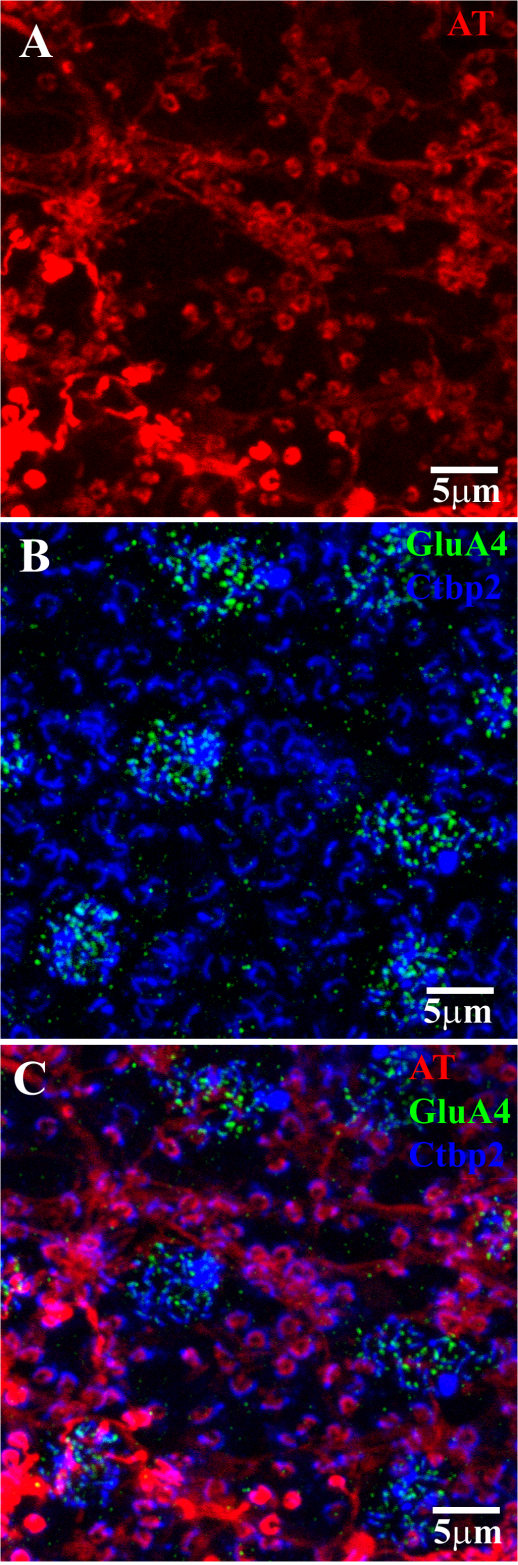
H1 axon terminal (AT) coupling with GluR4 and RIBEYE. **(A)** After injecting Neurobiotin into a single AT, a network of coupled ATs was revealed (red). The absence of HC somas indicated that the labeled network consisted only of AT endings, which appeared as a series of horseshoe-shaped profiles. **(B)** Ctbp2 (blue) labels the synaptic ribbons of both the cone pedicles and rod spherules. The GluA4 labeling shows the position of several cone pedicles within this field. The cone pedicles contained multiple synaptic ribbons, whereas the rod spherules contained only one large horseshoe-shaped ribbon. Double labeling with GluA4 (green) and RIBEYE can be used to map the rod–cone mosaic in the whole-mount retina. **(C)** A triple-label image shows that the H1 AT endings lie inside and concentric with the rod spherule synaptic ribbons (blue). This shows that the H1 ATs contact only rods. Note that there is an H1 AT ending at every rod spherule, which indicates the spread of Neurobiotin through the coupled network of the H1 ATs. Scale bar = 5 μm. Axon terminal (AT).

By directly injecting Neurobiotin into the ATs, without MFA, the entire primate H1 AT network could be stained (**Figure 4A**). This revealed a complex structure with no labeled somas, suggesting that Neurobiotin labeled the AT matrix only and that H1 somas and ATs form distinct networks. As other researchers have observed, the individual AT endings were horseshoe or question mark shaped, and only one horseshoe-shaped AT was visible per rod terminal.

RIBEYE is a component of the synaptic ribbon and labels synaptic ribbons in cone pedicles and rod spherules. The cone pedicles contain multiple fine ribbons, whereas rods contain one large horseshoe-shaped ribbon (**Figure 4B**). GluA4 antibodies were also used to label AMPA receptors in the clusters located beneath each cone pedicle. Double labeling with GluA4 and RIBEYE can be used to map the rod–cone mosaic in the whole-mount primate retina. The H1 AT network, labeled by the injection of Neurobiotin, did not contact cone pedicles. Instead, the horseshoe-shaped AT endings entered every rod spherule, and this was shown by the presence of a large synaptic ribbon. The AT processes occur closely and intimately inside and concentric to the rod synaptic ribbons, as previously reported for rabbit retina (47) (**Figure 4C**). Thus, we confirm that primate H1 ATs contact rod spherules exclusively.

### H2 horizontal cells

In primate retinas labeled with DAPI, the somas of the H2 HCs were smaller and dimmer than those of H1 HCs, with an average area of 66.5 µm^2^ ± 1.7 µm^2^ (mean ± SEM; *n* = 27). Neurobiotin injection into a single H2 HC soma revealed that these cells are highly coupled and axon-bearing.

**Figure 5:**
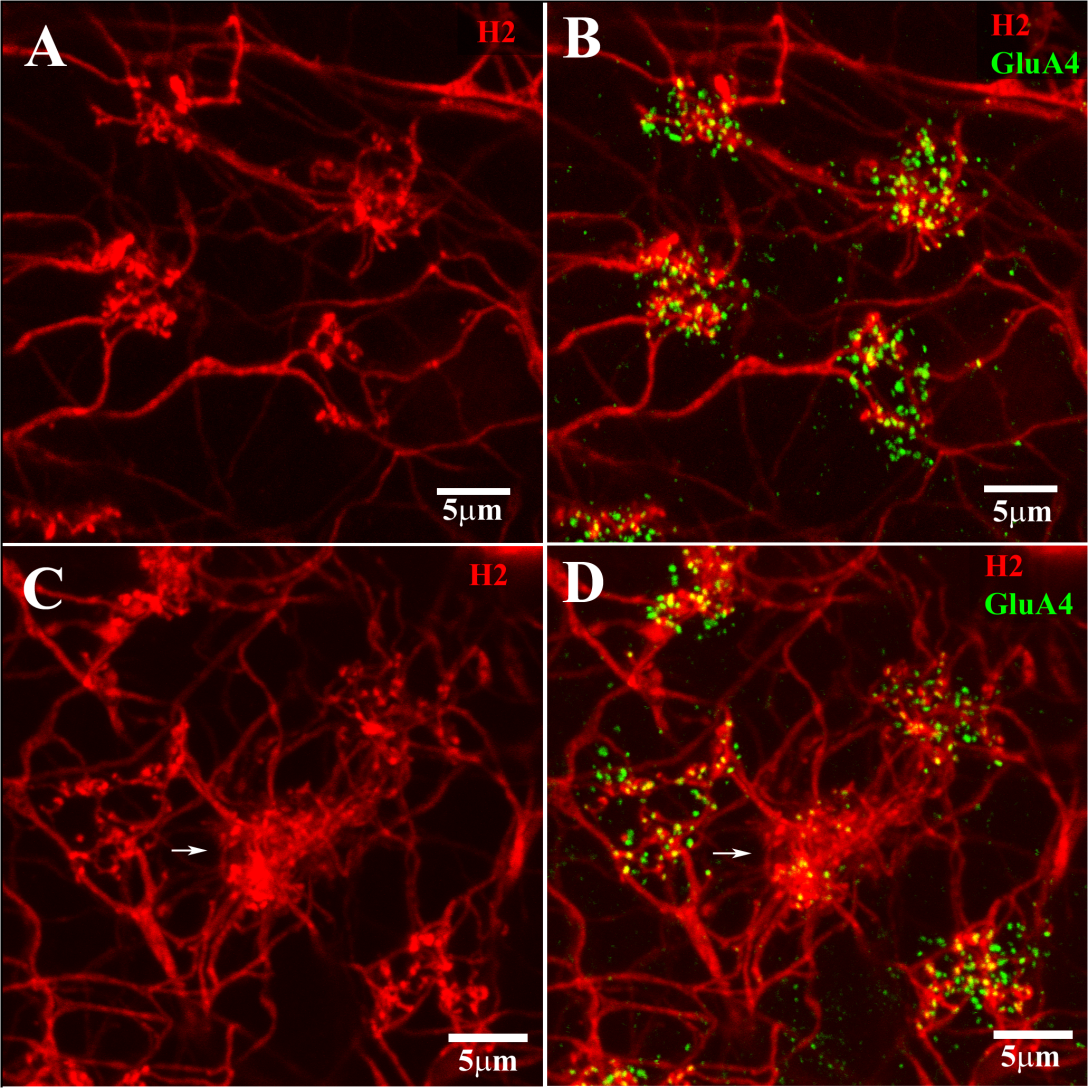
H2 coupling and cone contacts. This shows part of a Neurobiotin-filled patch of coupled H2 HCs. The H2 coupling was visualized using streptavidin conjugated with Alexa-Cy3 (red). The GluA4 receptor subunits are labeled in green. **(A, B)** show a portion of the H2-coupled network, with sparse clusters of terminals at four red/green cone pedicles, labeled for GluA4. **(C, D)** show a different portion of the same H2 network with sparse dendritic contact at four red/green cone pedicles and a dense cluster of H2 dendrites at one blue cone pedicle (center, white arrow). The dendritic terminal clusters at cone pedicles are of two different types. The dense dendritic cluster (white arrow) contacted a blue cone. The sparse dendritic cluster contacted the red/green cones. Scale bar = 5 μm. H2 horizontal cells (H2 HCs).

The H2 HC dendrites formed two kinds of clusters at cone pedicles: a dense cluster that contacted blue cones and a sparse cluster that contacted red/green cones (**Figure 5**). Many more H2 dendrites converged at each blue cone pedicle. Thus, the H2 HCs are primarily connected to blue cones, but only sparsely connected to red and green cones, as previously reported (15, 16, 49). This connectivity pattern is the opposite of that of the H1 HCs, which contact nearly all cones, but have few or no contacts at the blue cones.

**Figure 6:**
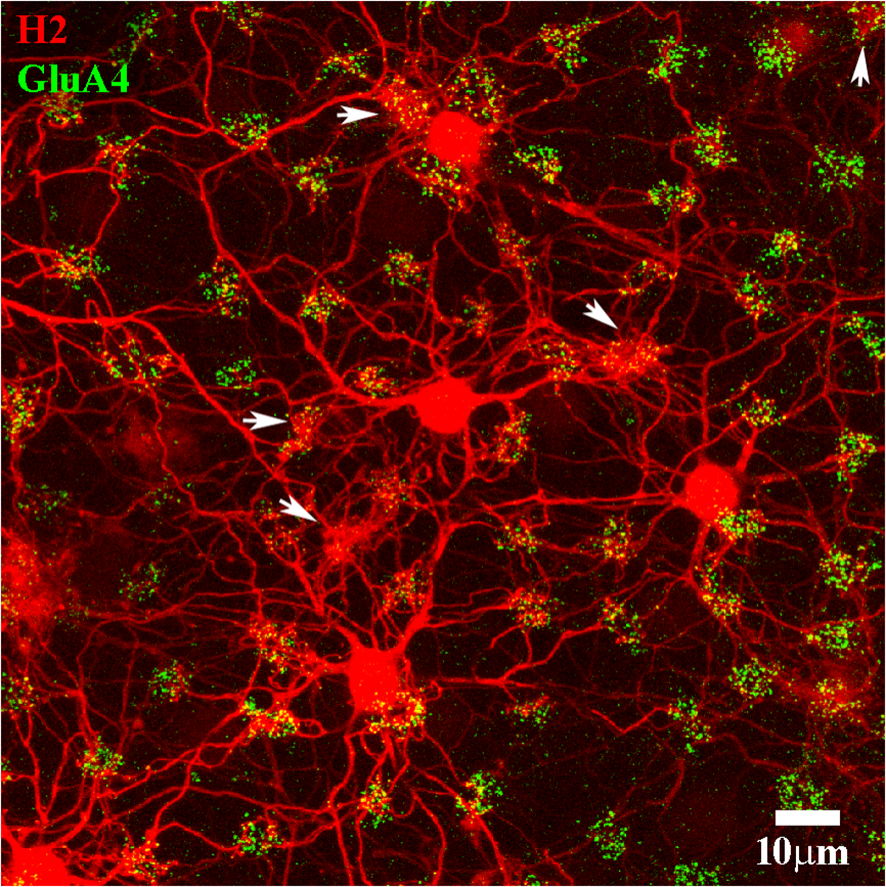
H2 cone contacts. A Neurobiotin-filled patch of H2 HCs (red). The H2 HCs are extensively coupled, and the center of this patch is out of the frame to the left. The cone pedicle array is marked with antibodies against GluA4 subunits (green). Note that the H2 network contacted almost every cone pedicle. There are six blue cone pedicles, marked with white arrows, noticeable by the dense convergence of H2 dendrites and the numerous dendritic contacts. The remaining red/green cones were nearly all in contact with the H2 dendrites. Scale bar = 10 μm. H2 horizontal cells (H2 HCs).

Although H2 dendrites make distinct and obvious connections with blue cone pedicles, it should be emphasized that H2 HCs contact nearly every cone. In **Figure 6**, a Neurobiotin-labeled patch of H2 HCs is shown. The positions of six blue cones are marked by arrows. This example was double-labeled for GluA4 subunits, which are concentrated at each pedicle and thus serve to tag the cone array. The H2 network contacted almost every cone pedicle in the frame. Of the 70 cone pedicles in the frame, 64 were red/green cones, and all except one were contacted by the H2 HC network. The red/green cone contacts of H2 HCs were sparse compared with the convergence of dendrites at the blue cone pedicles. The GluA4 subunits below each red/green cone, at the level of the desmosome-like contacts between HC dendrites, show as single-labeled green profiles because they are located mostly on the H1 dendrites, which are predominant at red/green cones. At the blue cone pedicles, the GluA4 subunits are double labeled with the H2 matrix and thus appear yellow.

### H2 axon terminals

**Figure 7:**
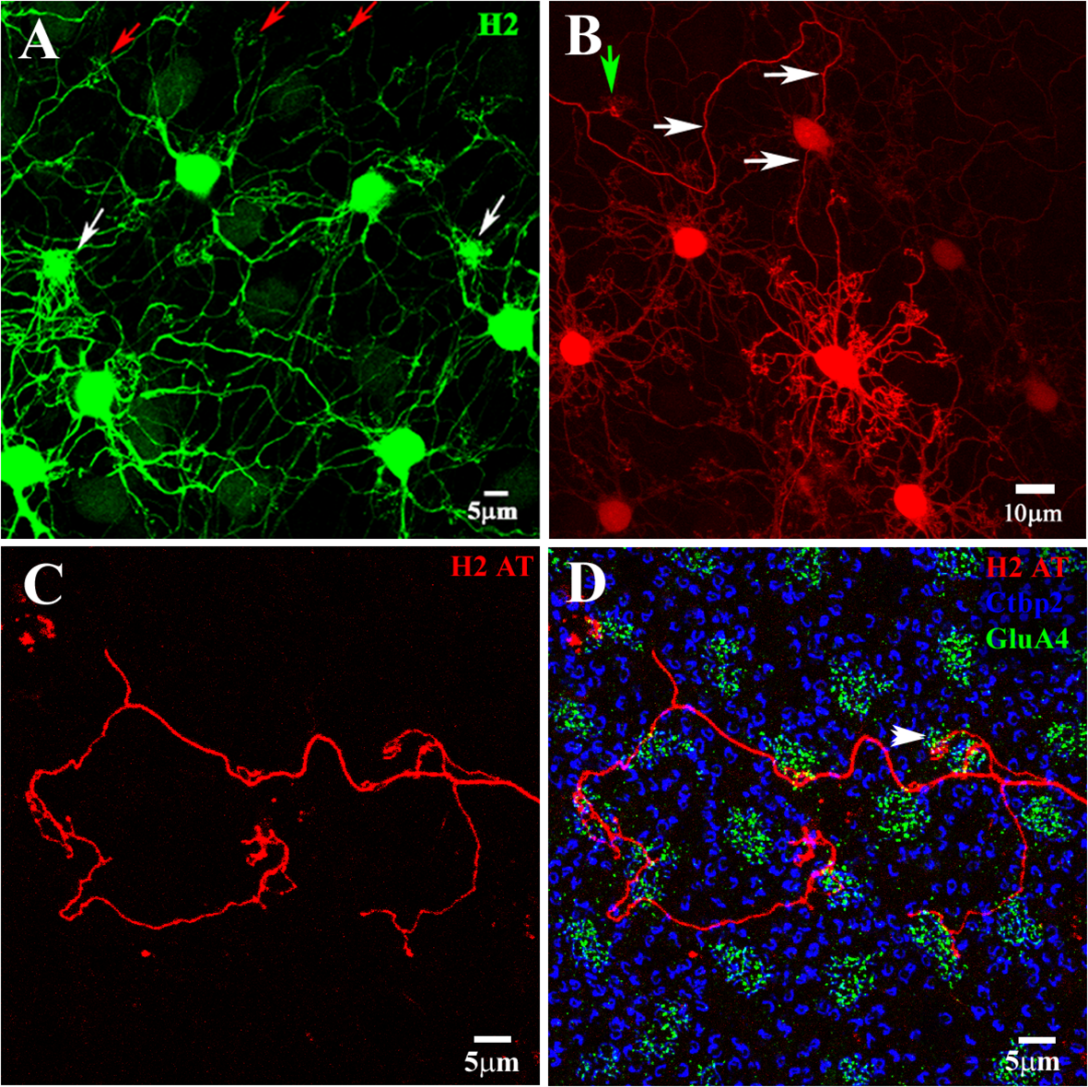
H2 horizontal cells. The H2 HCs were dye-injected with Neurobiotin and visualized with streptavidin–Alexa Fluor 488 **(A)** or streptavidin–Cy3 **(A, B, D)**. **(A)** The H2 HCs made two types of contacts with the cone pedicles. After injecting a single H2 soma, the H2 network was revealed (green). There were sparse groups of dendrites at some locations, corresponding to red/green cones (red arrows), and very dense clusters at a few locations, which corresponded to the blue cone pedicles (white arrows). Although the H2s had axons, they were poorly filled and lost in the dendritic network. H2 horizontal cells (H2 HCs). **(B)** In the presence of 100 uM MFA to reduce coupling, a single H2 HC was prominently filled, with clusters of dendritic terminals spaced to match the cone pedicle mosaic (green arrow). A single axon arose from this cell and meandered for several hundred microns (white arrows). **(C)** An H2 axon terminal (red) showing seemingly random progress and side branches. **(D)** In this triple-labeled material, it can be seen that the H2 AT (red) did not contact rods [marked by large synaptic ribbons (stained with Ctpb2, blue)]. In the center, a cluster of terminals that ended without a nearby cone did not contact the rod spherules, which were at a different level. The H2 AT contacted several cone pedicles (marked by clusters of GluA4 subunits, green), at least one of which was confirmed as a blue cone pedicle by its size, irregular position, and protrusion into the OPL (arrow). H2 axon terminal (H2 AT). **(A, C, D)** Scale bars = 5 μm. **(B)** Scale bar = 10 μm.

**Figure 8:**
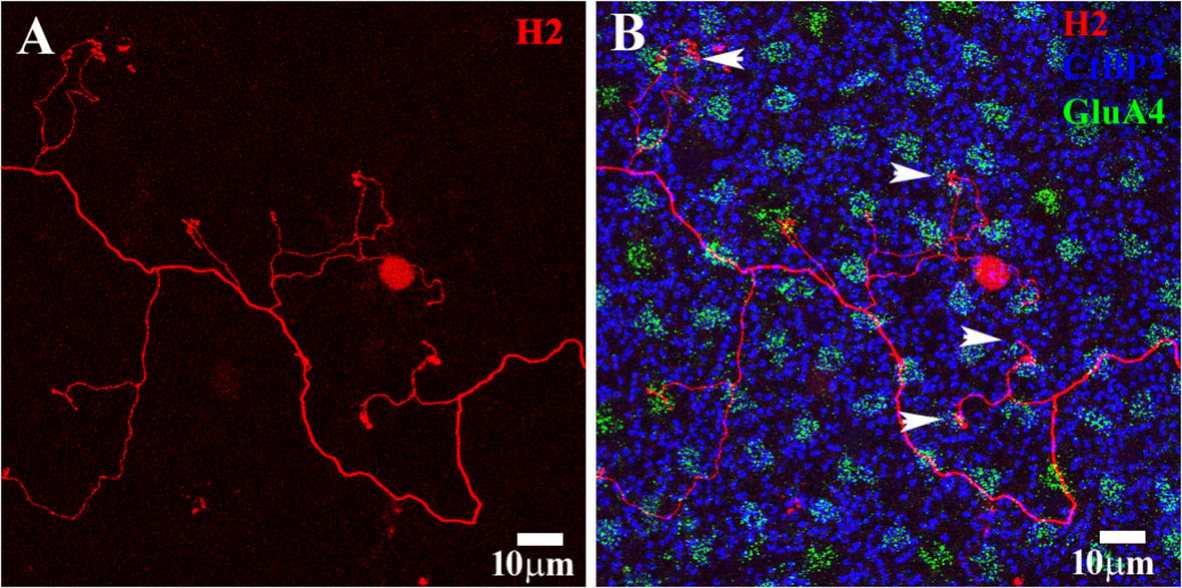
H2 axon terminal. **(A)** A Neurobiotin-filled H2 axon terminal (red) with a typical meandering axon and a few side branches. **(B)** The photoreceptor mosaic was stained using antibodies against GluA4 to show cone pedicles (green) and Ctpb2 to stain synaptic ribbons (blue). The rod spherule position is shown by large synaptic ribbons. The H2 AT contacted several cone pedicles. Four of them were identified as blue cone pedicles on the basis of their irregular position, which was adjacent to a red/green cone, and protrusion into the OPL. The H2 ATs did not contact rods. Scale bars = 10 μm. H2 horizontal cell (H2 HC).

The H2 HCs are also axon-bearing (**Figures 7B, C**), although we were able to recover only a few examples. Filling an H2 with Neurobiotin in the presence of 100 µM MFA to reduce the spread of tracer through the network of coupled H2 somas revealed an axon that meandered for several hundred microns in a random direction (**Figures 7B-D**). In another example, a single H2 AT was stained using 100 µM MFA (**Figure 8**). It is immediately obvious that the H2 AT axonal structure is quite different from that of the H1 AT. The H1 ATs bear a compact AT expansion which contacts most rods within the AT field. This is the same as the pattern that was observed in B-type HCs derived from rabbits (47).

In contrast, H2 axons did not elaborate into a single branching structure. Instead, they were found to have several poorly developed terminal branches that contact cones exclusively (**Figures 7**, **8**). At least one of the cones contacted by the H2 AT (arrow in **Figure 7D**) is smaller and adjacent to another cone pedicle and does not fit the cone mosaic. On this basis, it was identified as a blue cone pedicle (42). The H2 ATs do not contact rods. In the center of **Figures 7C, D**, a group of end terminals that are not associated with a cone is shown. Although it appears to be close to several rod spherules, focusing through the confocal series shows it is distinctly below the rod spherules and does not contact them. The target of this structure, if any, is unknown.

**Figure 8** shows another example of a H2 AT. Again, there was no major terminal expansion observed. Instead, several minor branches targeted cone pedicles, but there was no indication of rod contact. Several of the cone pedicles in contact with the H2 AT, marked by the accumulation of GluA4 labeling, were irregular with regard to the cone mosaic, often immediately adjacent to blue cones (arrowheads, **Figure 8B**). These putative blue cone pedicles also protruded into the OPL and had a slightly different plane of focus. We judged these pedicles to belong to blue cones, based on these properties, as described by Anhalt et al. (42). These results confirm that the H2 ATs contact cones, and exhibit some preference for blue cones (17). However, the function of the H2 ATs is unknown.

### Cone input to the H1 HCs was mediated by AMPA receptors

**Figure 9:**
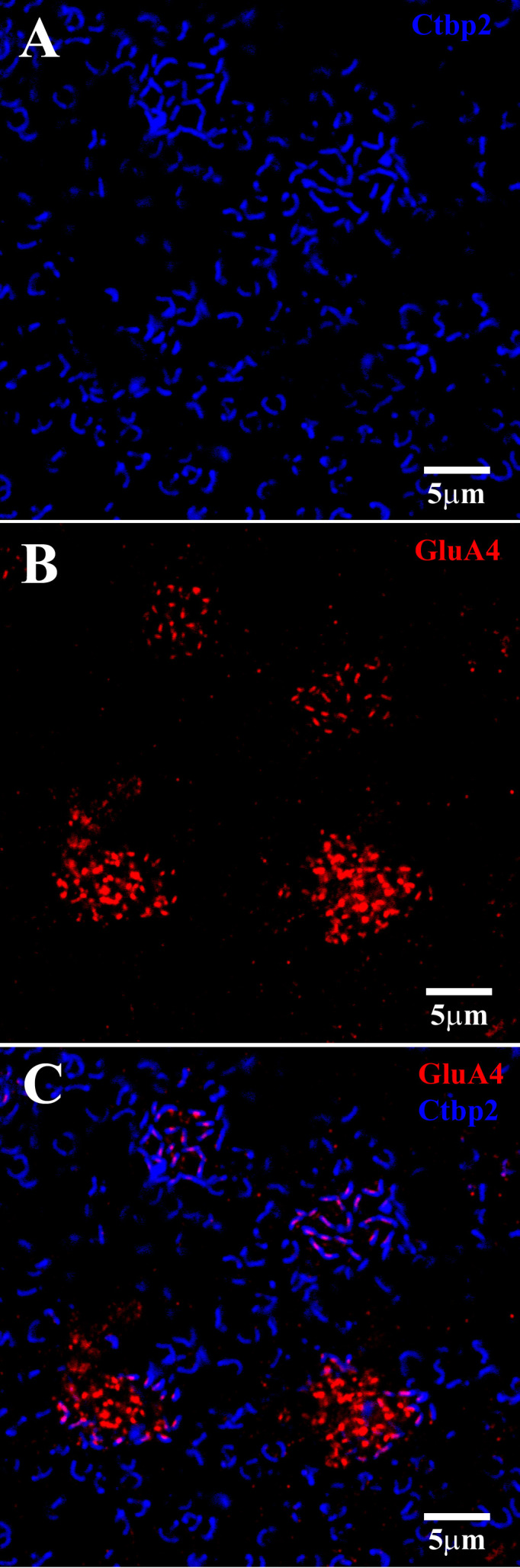
GluA4 subunits at cone pedicles. A group of four cone pedicles in the OPL. The plane of focus was slightly oblique, so that for the upper two cones, the focal plane was at the level of the synaptic ribbons, whereas for the lower two cones, the GluA4 subunits beneath the cones were in focus. **(A)** Upper two cones: Ctbp2-labeled synaptic ribbons (blue) at each cone pedicle and rod spherule. Cone pedicles contained a cluster of Ctbp2-labeled synaptic ribbons, whereas rod spherules had larger single ribbons with a question mark- or horseshoe-shaped structure. **(B)** The plane of focus was oblique. The GluA4 labeling (red) here shows two locations. The top two clusters were slightly higher, close to the synaptic ribbons of the cones. Note that the GluA4 subunits were small, orderly, and discrete. At the lower level of focus, approximately 1.5 μm below the cone pedicles, the red GluA4 signal was brighter, clustered, and disorganized. **(C)** Double-labeled image shows that GluA4 labeling (red) close to the cone pedicles, is registered with the synaptic ribbons (blue), such that there was one centrally located GluA4 cluster for each synaptic ribbon. At the lower level of focus, which was on the bottom two cones, the GluA4 subunits were denser but not aligned with the overlying synaptic ribbons. Scale bars = 5 μm.

The synaptic ribbons within cone pedicles and rod spherules were labeled with antibodies against RIBEYE (**Figure 9A**). The cones contained a group of fine, elongated ribbons, whereas the rod spherules enclosed a single, large, question mark- or horseshoe-shaped structure. The GluA4 subunits were located at two slightly different levels relative to the cone pedicles (**Figure 9B**) (20). The confocal frame in **Figure 9** is slightly oblique, such that the plane of focus for the top two cones is at the base of the pedicle, with the synaptic ribbons in focus. In this study, the GluA4 subunits were slim and lightly labeled, forming a regular mosaic in register with the synaptic ribbons (**Figure 9B**, the top two cones). The lower GluA4 site was found approximately 1.5 µm below the base of the cone pedicle (**Figure 9B**, bottom area), meaning that the cluster of cone synaptic ribbons was out of focus. At this lower site, the GluA4 labeling was bright and dense, appearing in the form of clusters that were not aligned with the synaptic ribbons. The glutamate receptors at this lower site have previously been described as occurring at desmosome-like contacts among HC dendrites (20, 37).

**Figure 10:**
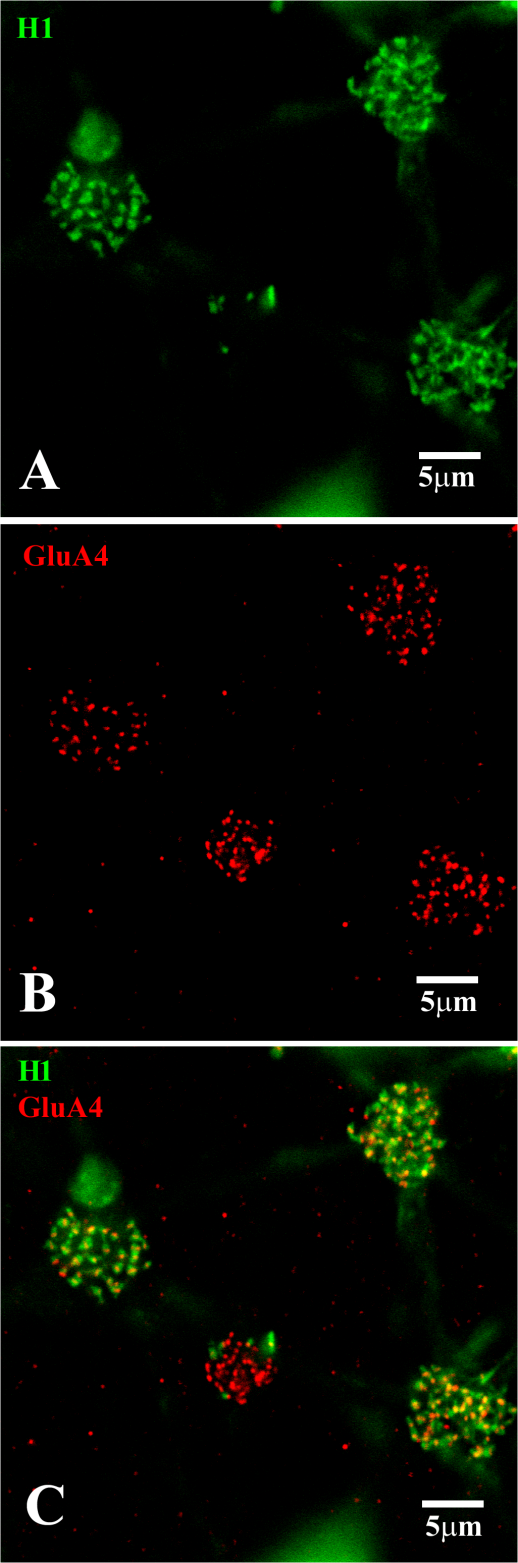
H1 dendrites have many contacts at red/green cone pedicles. **(A)** Neurobiotin-labeled H1 HC dendrites (green), with a focus on the cone pedicles. The H1 dendrites from several cells converge at each cone pedicle. The left-hand cone had very regular dendrites, whereas the two right-hand cones had dense and overlapping H1 dendrites at a level slightly below that of the cone pedicles. The central cone had only 4 or 5 H1 contacts, identifying it as a blue cone pedicle. **(B)** GluA4 (red)-labeled cone pedicles. For the left-hand cone, the GluA4 subunits were small and regular, showing the focal plane was close to the synaptic ribbons. For the other three cones, the focus was a little underneath each pedicle and the GluA4 subunits were dense and disorganized. The small size of the central cone pedicle indicated it was a blue cone. The other three red/green cones had larger pedicles. **(C)** Double-label image showing that clusters of GluA4 subunits (red) were colocalized with H1 dendrites (green) at two sites. At the tips of the H1 HCs dendrites, close to the synaptic ribbons, for the left-hand cone pedicle, every H1 dendrite was colocalized with GluA4 subunits. For the two right-hand cones, the focus was just beneath the pedicles, where there was a dense and irregular cluster of GluA4 staining, colocalized with irregular and overlapping H1 dendrites. The smaller blue cone pedicle had many GluA4 subunits but very few H1 contacts. Scale bars = 5 μm. H1 horizontal cells (H1 HCs).

We also processed dye-injected material to determine directly if the dendrites of HCs expressed glutamate receptors. **Figure 10** shows the terminal clusters of the H1 HCs at four different cone pedicles. For the left-hand cone, the plane of focus is at the base of the cone pedicle, in which the GluA4 subunits formed a small regular grouping. At this cone, the H1 dendritic terminals were discrete, and the GluR4 labeling was aligned with the tips of the H1 dendrites. Every H1 dendrite was aligned with a glutamate receptor. For the two right-hand cones, the plane of focus was just underneath the pedicles. At this level, a bright yet irregular cluster of GluA4 subunits was colocalized, with many overlapping H1 dendrites observed beneath the cone pedicles. Finally, the middle cone had a smaller cluster of GluA4 subunits and very few H1 HC dendritic terminals. The smaller size and the small number of H1 contacts indicated that this cone pedicle belonged to a blue cone.

**Figure 11:**
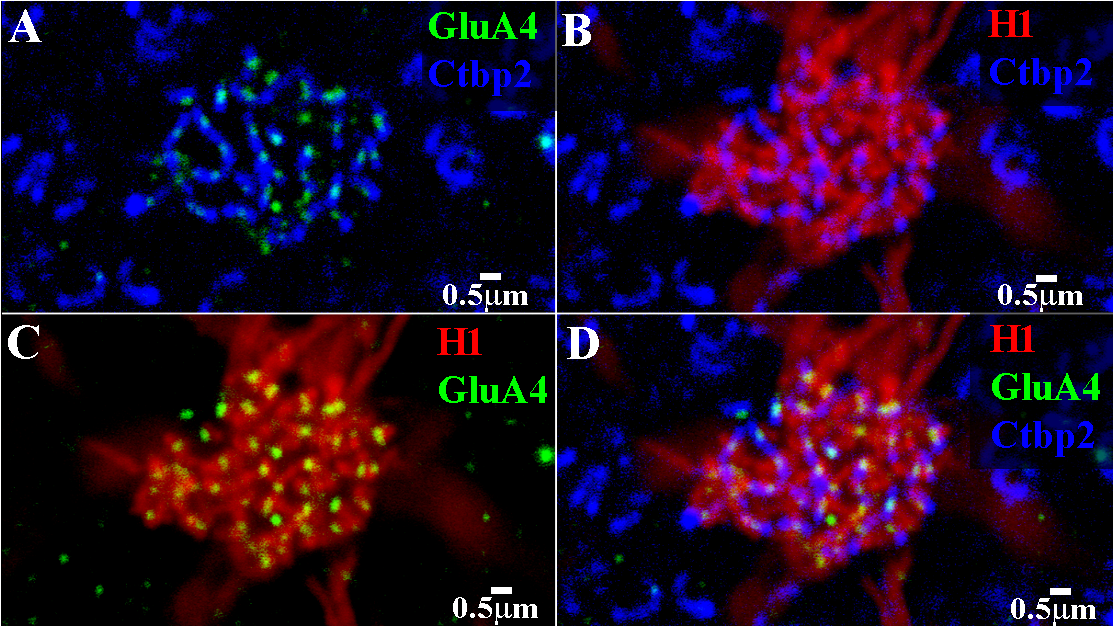
Triple-label image showing H1 contacts with a red/green cone pedicle. High-resolution image of a single cone pedicle, a mini-stack of 3 μm × 0.27 μm sections, focused on the synaptic ribbons. **(A)** The synaptic ribbons, stained for Ctbp2 (blue), formed a discrete, non-overlapping network. The GluA4 subunits (green) were located on every ribbon, usually in the middle of the ribbon. **(B)** H1 dendrites (red) approached closely to nearly every synaptic ribbon (blue). The dense H1 contacts identified this as a red/green cone. **(C)** The H1 dendrites (red) were colocalized with the GluA4 subunits (green). Every H1 dendrite contained a GluA4 cluster. However, there were a few isolated GluA4 subunits (top left). Presumably, these positions were occupied by unlabeled H2 dendrites. **(D)** Triple-labeled image that shows that the H1 dendrites (red) and GluA4 subunits (green) were closely and stereotypically associated with the cone synaptic ribbons (blue). Normally, there are two H1 dendrites flanking the GluA4 subunits at each ribbon. This indicated that the H1 HCs received cone input mediated by AMPA receptors. A few synaptic ribbons (top left) were associated with GluA4 subunits, but these positions had no H1 contacts. Presumably, these positions were filled by H2 contacts, which also express GluA4 subunits. Scale bars = 0.5 μ.r. H1 horizontal cell (H1 HC).

A high-resolution image of a single cone pedicle is shown in **Figure 11**. The focus was on the level of the synaptic ribbons at the base of the cone pedicle. There are a large number of synaptic ribbons (≈ 32). At this level, the GluA4 subunits were in register with the synaptic ribbons and every synaptic ribbon was associated with a GluA4 cluster, usually in the middle of the ribbon. The dendritic tips of the H1 HCs approached very closely to the synaptic ribbons. There were H1 dendrites at most synaptic ribbons, which identified this as a red/green cone pedicle. A double-label image showed that every H1 dendrite was colocalized with a GluA4 cluster. However, there are a few isolated GluA4 subunits, presumably occupied by unlabeled H2 dendrites, which have fewer contacts with red/green cones. Finally, in the triple-label image, it was perhaps more obvious that laterally placed H1 dendrites flanked the GluA4 cluster at nearly every ribbon. This is the confocal appearance of a classic triad (18, 46).

Although the GluA4 antibody very clearly labeled the cone mosaic, there was also faint and sporadic labeling among the cone pedicles. Unfortunately, these signals were too weak and unreliable for analysis. Undoubtedly, some of the signals were non-specific or background labeling, but some appeared to be associated with the rod spherules. We note that the small glutamate receptors have been located at both HC and OFF bipolar cell contacts with rods in other mammalian species (30, 47, 50).

### Cone input to the H2 HCs was mediated by AMPA receptors

The arrangement of H1 dendritic contacts and H2 dendritic contacts with the various types of cones was quite different. It has been shown that H2 dendrites make more contact with blue cones than they do with red/green cones (15, 16, 49). In fact, the location of blue cone pedicles can be identified by the confluence of many H2 dendrites at one position (**Figures 5C**, **6**). Although H2 dendrites contacted every cone pedicle, there were relatively few contacts at red/green cones. At the blue cones, the numerous H2 dendrites aligned with the ribbons and GluA4 subunits, as did the less numerous H2 contacts at red/green cones. As before, the GluA4 subunits were located at two different depths under each cone pedicle. Those GluA4 subunits close to the synaptic ribbons were in register with them, whereas the lower brighter GluA4 subunits had no obvious relation to the synaptic ribbons. The lower GluA4 subunits were colocalized with H2 dendrites at the blue cones, but at the red/green cones, they were mostly independent of H2 dendrites, being colocalized with H1 dendrites, which were predominant at the red/green cones. In summary, the presence of GluA4 subunits at the H2 dendritic contacts close to the synaptic ribbons indicates that cone signaling to the H2 HCs is mediated by AMPA receptors. It would appear that the GluA4 receptor subunit is used for both HC types at red/green and blue cone pedicles.

**Figure 12:**
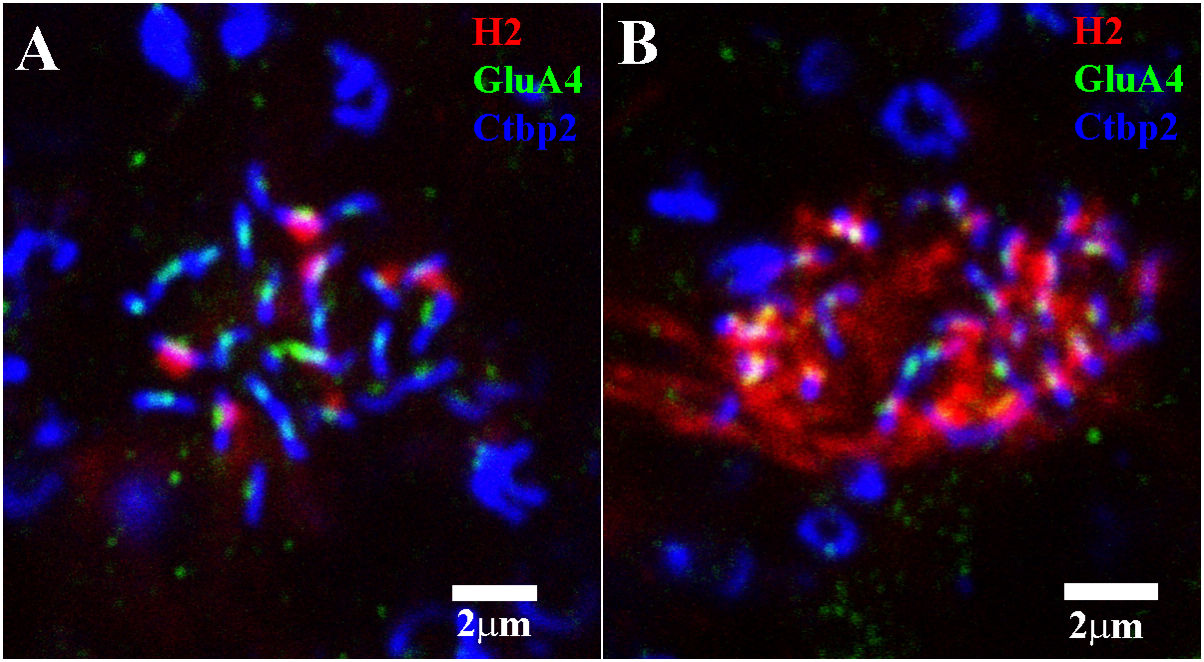
Triple-label image showing H2 HC contacts with red/green cone pedicles and blue cone pedicles. **(A)** In this red/green cone pedicle, a mini-stack of 3 μm × 0.30 μm sections, which was focused on the synaptic ribbons, there were few H2 (red) contacts. When they did occur, they took up one side of a pair at each ribbon (blue)/GluA4 (green) subunit. Presumably, the other side and all the empty ribbons were occupied by H1 dendrites. **(B)** At this blue cone pedicle, there are many more H2 dendrites (red). Nearly every ribbon (blue)/GluA4 (green) site was occupied by H2 HC dendrites (red), but there were a few empty positions, presumably filled by H1 dendrites. Scale bars = 2 μm. H2 horizontal cell (H2 HC).

**Figure 12A** depicts a high-resolution view of the H2 dendritic tips at a single red/green cone pedicle, triple labeled for H2 dendrites, GluA4 subunits, and RIBEYE to visualize the synaptic ribbons. The green GluA4 subunits are located centrally, just below the middle of every ribbon. There were only a few H2 dendrites (seven, to be exact) at the red/green cone pedicle, which were located laterally to one side of each ribbon at the GluA4 site. Typically, there are two HC elements on either side of the synaptic ribbon, visible in the H1 contacts with red/green cones as an equals sign with the ribbon in the middle (18). The H2 labeling pattern at the red/green cones suggests that (i) most empty synaptic ribbons are contacted by H1 HC dendrites, with a pair at each ribbon; and (ii) in cases in which a single H2 contact occurred, the other unlabeled lateral site was occupied by a H1 dendrite. Thus, a few synaptic ribbons were flanked by an H1/H2 pair of dendrites.

The H2 HC contacts at a single blue cone pedicle are shown in **Figure 12B**. Clearly, there are many more H2 dendrites than H1 dendrites at blue cone pedicles, but there are still a few empty positions, presumably filled by H1 dendrites, which are unlabeled. Every H2 dendrite was colocalized with a GluA4 subunit at the synaptic ribbon.

### Cone input to the H1 and H2 networks

Using the Neurobiotin-filled primate HCs, we attempted to count the numbers of H1 and H2 contacts at the red/green and blue cone pedicles. It was a simple matter to count the minority contacts at each cone type, that is, the number of H1 contacts with the blue cones and the number of H2 contacts with the red/green cones. However, we could not count the majority of contacts reliably because of the overlap among dendrites caused by crowding and the limited resolution (see also 16) (16). Instead, we estimated the number of majority HC contacts by counting the total number of ribbons and/or HC dendritic clusters. Presuming there are two lateral HC contacts at each synaptic ribbon (18), we multiplied the number of ribbons by two and then subtracted the number of minority contacts to calculate the number of majority contacts.

For cones at a mid-peripheral location, as were used in this study, there were 27.2 ± 0.7 (mean, ± SEM; *n* = 9) ribbons to one red/green cone pedicle, and 25.6 ± 0.7 (mean ± SEM; *n* = 9) ribbons to one blue cone pedicle. As previously reported, red/green and blue cone pedicles have a similar number of synaptic ribbons (21, 42). At the red/green cones, there were few H2 contacts, namely 6.5 ± 0.4 per cone (mean ± SEM; *n* = 7) out of 54 potential sites, presuming there were two flanking HC sites per ribbon (**Figure 12A**). Subtracting the H2 contacts suggests there were approximately 48 H1 contacts per red/green cone (**Figure 11**). At the blue cones, there were a similar number of ribbons with 3.8 ± 0.5 (mean, ± SEM; *n* = 6) H1 contacts (**Figure 10**), leaving 51 − 3.8 = approximately 47 H2 contacts per blue cone pedicle (**Figure 12B**). Owing to the small number of blue cones with a few H1 contacts, the blue cone input to H1 HCs was very minor, and the red/green cone input to the H1 network was dominant. But the small number of H2 contacts at the most numerous red/green cones, makes a large red/green input to the H2 network. Thus, the H2 HCs receive mixed inputs (15).

In one of our mid-periphery samples, blue cones constituted 8.1%, and red/green constituted 92% of the total number of cones, which was consistent with previous reports (42). Weighting the number of contacts by the type of cone input, we estimate that the red/green cone contacts with the H1 HCs constitute 99.3% of the total cone input, and that the sparse blue cone contact with the H1 HCs made up only 0.7% of the total cone inputs. Thus, it is not surprising that a blue stimulus evokes a very minor or negligible response from the H1 HCs; they are driven almost exclusively by the red/green cones, which is consistent with the weighted input we have shown here (15). In contrast, the sparse contact of the more numerous red/green cones constituted 58% of the cone input to the H2 HCs, and the dense blue cone contacts with the H2 HCs made up 42% of this cone input. This matches the mixed spectral response of the H2 HCs, which have strong responses to red, green, or blue stimuli, in line with their mixed dendritic contacts (15).

## Discussion

Our results may be summarized as follows: in the primate retina, there are two distinct types of HC, known as H1 and H2 cells. Both cell types have AT structures. Although the H1 ATs contact rod spherules, we confirmed that the H2 ATs contact cones, with some preference for blue cones. There are at least three independently coupled networks—H1 somas, H2 somas, and H1 ATs. We can confirm that H1 HCs have many contacts with the red/green cones, yet only a few contacts with the blue cones. Conversely, H2 has many contacts with the blue cones and although they also contact most red/green cones, they have fewer contacts with these cones. The cone contacts of H1 and H2 HCs are consistent with their spectral responses (15). The dendritic tips of H1 and H2 expressed GluA4, in register with the cone synaptic ribbons. This indicates that cones drive both H1 and H2 HCs via AMPA receptors.

### Horizontal cells formed at least three coupled networks

We have injected the gap junction permeant neuronal tracer Neurobiotin into the HC networks of the primate retina. We can confirm that the primate retina has two morphological types of HC, both axon-bearing. The dye injection of H1 HCs produced a dense network of coupled cells, whereas injecting H2 HCs produced a similar second network with fewer dendrites. In both cases, axons could be observed but not followed to the AT. Blocking the HC gap junctions with MFA revealed the AT structures. The H1 axons expanded into elaborate structures with many fine endings, exclusively contacting rod spherules. Therefore, primate H1 HCs appear to be analogous with the B-type HCs of the rabbit retina and the HCs of the mouse retina. The H2 ATs were morphologically distinct and they had sparsely branched axons with several collaterals. In contrast to the H1 ATs, the sparse contact of H2 ATs was made with cone pedicles, confirming the findings of a previous study by Chan and Grünert (17). The axons of primate HCs were very long, meaning that a high dose of MFA was required to isolate and follow them. We observed one H1 axon that was 350 um, and H2 axons of more than 200 um in length, before staining faded. These observations are consistent with those made in a previous study, which showed that HC axons in the primate retina were between 1.5 mm and 2.5 mm in length (43).

When facilitating the recovery of single-cell structure, the use of MFA obviously precludes the ability to observe AT coupling. However, the H1 AT matrix is dense and it is possible to inject H1 ATs blindly at the correct electrode depth without MFA. Under these circumstances, a coupled network of H1 AT processes contacting all rod spherules was observed. In rabbits, the single B-type ATs contact only 10% of the rod spherules within the AT field (47). In primate retinas, the H1 AT contacts with every rod spherule indicated that multiple H1 ATs were filled with a single injection. In other words, H1 ATs were dye-coupled. Thus, there are three independently coupled HC networks in the primate retina: H1 dendrites, H2 dendrites, and H1 ATs. Presumably, gap junction coupling expands the receptive field of each cell type (15, 51). The H2 ATs could not be targeted or stained without MFA, and the sparsely branched structures were not amenable to dye injection. Thus, we could not determine if the H2 ATs were independently coupled.

We noted that the dose of MFA required to block H1 dendritic coupling to reveal the AT structure was higher than that previously used in the rabbit retina (29). This could mean that primate HCs are better coupled, but it is more likely that MFA is less effective in the primate retina or that the thickness of the primate retina impedes penetration and effectively lowers the concentration of MFA in the OPL. Paired recordings to measure the gap junction conductance between adjacent HCs may be required to resolve this matter.

Electrical coupling is mediated by gap junctions composed of connexins; however, the specific connexins expressed by primate HCs are unknown. In general, Cx36 is the most common neuronal connexin and it is widely expressed in the retina (52). However, HCs do not express Cx36. The single HC type in the mouse retina expresses Cx57 (53, 54). In the rabbit retina, A-type HC gap junctions are labeled for Cx50, and the B-type AT network is labeled for Cx57 (55, 56). In fish retina, HCs express several connexins from a group with molecular weights around 50 K (57). Thus, based on both mammals and fish, connexins with molecular weights greater than 50 K seem the most likely candidates to support coupling in primate HCs, but they have not yet been identified.

### HC connectivity and AMPA receptors

GluA4 subunits were located on the dendritic tips of both H1 and H2 dendrites, very close to and aligned with the synaptic ribbons of both the red/green cones and blue cones. This direct demonstration of GluA4 expression, juxtaposed with the synaptic ribbons, indicated that all cone pedicles drive both H1 and H2 HCs via AMPA receptors. Previously, Haverkamp et al. (20, 37) showed clearly through immuno-EM that both HC processes at each triad express AMPA receptors in primate retina. It is known that an individual HC contributes only one process at each triad, and the other HC process must arise from a different HC (17, 45, 51). Although previously, researchers have been unable to identify H1 and H2 dendrites in the EM material, they deduced that if GluA4 was expressed in HC dendrites at every ribbon at a red/green cone, this indicated that the H1 HCs carry AMPA receptors. Similarly, they determined that if there were AMPA receptors on HC processes at every blue cone ribbon, then the H2 HCs must also express AMPA receptors (21). The AMPA receptors were also expressed on the dendritic tips of rabbit HCs (47). Finally, genetically removing AMPA receptors from mouse HCs caused a dramatic reduction in the cone-driven light response of mouse HCs (58). Thus, there is strong agreement from several lines of evidence that cones drive HCs via AMPA receptors.

The OFF bipolar cells in primate and mouse retinas are driven via kainate receptors of at least two types (38, 40). In the cone-dominated ground squirrel, several OFF bipolar cell types also use kainate receptors, but the OFF cb2 bipolar cell is driven by AMPA receptors (39, 59). Interestingly, the cb2 bipolar cell is triad-associated, meaning it is partly invaginating, and its dendritic tips approach the cone synaptic ribbons. This is different from the kainate-driven OFF bipolar cells, which terminate at a greater distance from the synaptic ribbon, often making synaptic contacts at the base of the cone pedicle relatively distant from the synaptic ribbons (59). In general, the postsynaptic arrangement at mammalian cone pedicles is complex, with as many as 15 postsynaptic cell types and several hundred postsynaptic processes converging at each cone pedicle (18).

The glutamate release sites lie at the cone synaptic ribbons (60). From here, the glutamate concentration is diluted and spread by diffusion, and the most distant processes incur a significant delay (61). Thus, the postsynaptic response is influenced by the glutamate receptor type, auxiliary proteins, the affinity for glutamate, and the distance from the synaptic ribbon. The cb2 bipolar cell dendrites express AMPA receptors and because they are triad-associated, lie close to the synaptic ribbon. The light-driven responses of cb2 bipolar cells are extremely sensitive, with the ability to respond to a single synaptic vesicle fusion event, and they have high temporal fidelity (59). In contrast, the more distant OFF bipolar cells, which use kainate receptors, are unable to detect single vesicle events and respond in a non-linear fashion to multivesicular events.

The analysis of OFF bipolar responses is relevant to the position of AMPA receptors on HCs at the triads of primate cone pedicles. Of the many postsynaptic processes, the laterally placed HC dendrites are closest to the cone synaptic ribbons, and the AMPA receptors are aligned with the synaptic ribbons. Thus, it is likely that HC responses have both rapid and sensitive responses to cone input. In contrast, the location of the lower band of AMPA receptors is puzzling. They are large and bright but located some distance away, that is, 1.5 µm from the triads (**Figure 9**) (20, 37). Furthermore, they are not registered with the synaptic ribbons. They do not seem to be ideally suited to receive synaptic input from cones. Perhaps, similar to the more distant kainate-driven bipolar cells, which contact the cone pedicle base, they respond slowly to bulk glutamate release (59), though the HC AMPA receptors are even more distant from the glutamate release site.

### Cone postsynaptic structure

As we have demonstrated, there are GluA4 subunits at both H1 and H2 HC contacts with cone pedicles in the primate retina (21). Let us consider the postsynaptic arrangement of HC dendrites and glutamate receptors. The cone synaptic ribbons can be marked with an antibody against RIBEYE. There appears to be one AMPA receptor containing a GluA4 subunit at each ribbon, yet there are two laterally placed HC dendrites at each site. Perhaps this is most clearly seen in **Figure 12**, in which a few H2 dendrites contact a red/green cone pedicle, occupying one lateral position, whereas the second lateral position must be filled by an unlabeled H1 dendrite because H1 dendrites occupy the majority of positions at the red/green cones. The simplest explanation is that there are actually two GluA4 subunits, close to the ribbon, one for each HC dendrite (H1 and H2), which are smaller than the resolution limit of the confocal microscope. Due to the resolution limit (200 nm–300 nm), these two medially placed receptors merge and appear as one between the HC dendrites, just under the synaptic ribbon, as shown in immuno-EM studies (21). The ON bipolar cells, decorated with GRM6 receptors, occupy a slightly lower position. Discriminating between the two AMPA receptors may be an interesting test of super-resolution microscopy in future experiments.

### Balance of cone inputs to the H1 and H2 HC networks

We have shown that most HC contacts at red/green cone pedicles are made with H1 HCs, and, conversely, at blue cone pedicles, that most of the contacts go to H2 HCs. This confirms the findings in the previous work of several groups (15, 16, 49). These data are based on Neurobiotin-injected HCs, which provided well-coupled patches of HCs, reflecting the H1 or H2 network. If we assumed that the weight of cone contacts reflects the input from each spectral cone type, and we accounted for the relative number of red/green vs. blue cones, we could estimate the spectral input to each HC network.

In the mid-peripheral macaque retina used in this study, the red/green cones constituted most of the cones (92%), with blue cones constituting approximately 8% of the cones. Weighting the number of cone contacts with H1 and H2 HCs by the fraction of red/green or blue cones, we estimated that the red/green cones constitute 99.3% of the input to H1 HCs, whereas the sparse blue cone contacts with H1 HCs made up only 0.7% of the total. The H2 HCs receive a more balanced input whereby the sparse contact of the more numerous red/green cones constituted 58% of the cone input to H2 HCs, whereas the dense contacts with a few blue cones made up 42% of the cone input to H2 HCs. This matches the balanced spectral response of H2 HCs, with strong responses to red, green, or blue stimuli, in line with their mixed dendritic contacts (15).

Previously, Goodchild et al. (16) determined that the blue input to H1 HCs was 1.9% and 11% to H2 HCs. Our results are in broad agreement with these results but the increased resolution from the confocal analysis of dye-injected HCs produced larger numbers of dendritic contacts, particularly for the majority of contacts (red/green to H1 and blue to H2), and changed the calculation slightly, such that the balance of predicted cone input to H2 was pushed further toward the blue cones. The sparse input from a few blue cones to H1 HCs was negligible compared with the dense input from a large number of red/green cones, which were dominant. For H2 HCs, a few contacts with a large number of red/green cones were sufficient to provide a red/green input (58%) in addition to the signal from blue cones (42%). Thus, H2 HCs received a mixed spectral input with responses to red, green, and blue stimuli (15).

## Data availability statement

The raw data supporting the conclusions of this article will be made available by the authors, without undue reservation.

## Ethics statement

The animal study was approved by the Animal Welfare Committee at the University of Texas Health Science Center in Houston. The study was conducted in accordance with the local legislation and institutional requirements.

## Author contributions

FP contributed to the acquisition, analysis, and interpretation of data. FP and SCM contributed to the conception and design of the work and contributed to the drafting of the manuscript. All authors contributed to the article and approved the submitted version.

## References

[1] Kamermans M Spekreijse H. The feedback pathway from horizontal cells to cones. A mini review with a look ahead. Vision Res (1999) 39:2449–68. doi: 10.1016/S0042-6989(99)00043-7 10396615

[2] Packer OS Verweij J Li PH Schnapf JL Dacey DM. Blue-yellow opponency in primate S cone photoreceptors. J Neurosci (2010) 30:568–72. doi: 10.1523/JNEUROSCI.4738-09.2010 PMC2826135 20071519

[3] Dacey DM Crook JD Packer OS. Distinct synaptic mechanisms create parallel S-ON and S-OFF color opponent pathways in the primate retina. Vis Neurosci (2014) 31:139–51. doi: 10.1017/S0952523813000230 PMC4309572 23895762

[4] Thoreson WB Dacey DM. Diverse Cell Types, Circuits, and Mechanisms for Color Vision in the Vertebrate Retina. Physiol Rev (2019) 99:1527–73. doi: 10.1152/physrev.00027.2018 PMC6689740 31140374

[5] Tsukamoto Y Iseki K Omi N. Helical Fasciculation of Bipolar and Horizontal Cell Neurites for Wiring With Photoreceptors in Macaque and Mouse Retinas. Invest Ophthalmol Visual Science (2021) 62:31. doi: 10.1167/iovs.62.1.31 PMC7846946 33507230

[6] Flores-Herr N Protti DA Wassle H. Synaptic currents generating the inhibitory surround of ganglion cells in the mammalian retina. J Neurosci (2001) 21:4852–63. doi: 10.1523/JNEUROSCI.21-13-04852.2001 PMC6762364 11425912

[7] Vanleeuwen MT Joselevitch C Fahrenfort I Kamermans M. The contribution of the outer retina to color constancy: a general model for color constancy synthesized from primate and fish data. Vis Neurosci (2007) 24:277–90. doi: 10.1017/S0952523807070058 17592668

[8] Chapot CA Euler T Schubert T. How do horizontal cells 'talk' to cone photoreceptors? Different levels of complexity at the cone-horizontal cell synapse. J Physiol (2017) 595:5495–506. doi: 10.1113/JP274177 PMC5556172 28378516

[9] Hirasawa H Kaneko A. pH changes in the invaginating synaptic cleft mediate feedback from horizontal cells to cone photoreceptors by modulating Ca2+ channels. J Gen Physiol (2003) 122:657–71. doi: 10.1085/jgp.200308863 PMC2229595 14610018

[10] Fahrenfort I Klooster J Sjoerdsma T Kamermans M. The involvement of glutamate-gated channels in negative feedback from horizontal cells to cones. Prog Brain Res (2005) 147:219–29. doi: 10.1016/S0079-6123(04)47017-4 15581709

[11] Vessey JP Stratis AK Daniels BA Da Silva N Jonz MG Lalonde MR. Proton-mediated feedback inhibition of presynaptic calcium channels at the cone photoreceptor synapse. J Neurosci (2005) 25:4108–17. doi: 10.1523/JNEUROSCI.5253-04.2005 PMC6724943 15843613

[12] Grove JCR Hirano AA De Los Santos J Mchugh CF Purohit S Field GD. Novel hybrid action of GABA mediates inhibitory feedback in the mammalian retina. PloS Biol (2019) 17:e3000200. doi: 10.1371/journal.pbio.3000200 30933967 PMC6459543

[13] Peichl L Gonzalez-Soriano J. Morphological types of horizontal cell in rodent retinae: a comparison of rat, mouse, gerbil, and Guinea pig. Vis Neurosci (1994) 11:501–17. doi: 10.1017/s095252380000242x 8038125

[14] Kolb H Mariani A Gallego A. A second type of horizontal cell in the monkey retina. J Comp Neurol (1980) 189:31–44. doi: 10.1002/cne.901890103 6766145

[15] Dacey DM Lee BB Stafford DK Pokorny J Smith VC. Horizontal cells of the primate retina: cone specificity without spectral opponency. Science (1996) 271:656–9. doi: 10.1126/science.271.5249.656 8571130

[16] Goodchild AK Chan TL Grünert U. Horizontal cell connections with short-wavelength-sensitive cones in macaque monkey retina. Vis Neurosci (1996) 13:833–45. doi: 10.1017/S0952523800009093 8903027

[17] Chan TL Grünert U. Horizontal cell connections with short wavelength-sensitive cones in the retina: a comparison between New World and Old World primates. J Comp Neurol (1998) 393:196–209. doi: 10.1002/(SICI)1096-9861(19980406)393:2<196::AID-CNE5>3.0.CO;2-Y 9548697

[18] Chun MH Grünert U Martin PR Wässle H. The synaptic complex of cones in the fovea and in the periphery of the macaque monkey retina. Vision Res (1996) 36:3383–95. doi: 10.1016/0042-6989(95)00334-7 8977005

[19] Massey SC. Chapter 11 Cell types using glutamate as a neurotransmitter in the vertebrate retina. Prog Retinal Res (1990) 9:399–425. doi: 10.1016/0278-4327(90)90013-8

[20] Haverkamp S Grünert U Wässle H. The cone pedicle, a complex synapse in the retina. Neuron (2000) 27:85–95. doi: 10.1016/s0896-6273(00)00011-8 10939333

[21] Haverkamp S Grünert U Wässle H. The synaptic architecture of AMPA receptors at the cone pedicle of the primate retina. J Neurosci (2001) 21:2488–500. doi: 10.1523/JNEUROSCI.21-07-02488.2001 PMC6762391 11264323

[22] Collingridge GL Olsen RW Peters J Spedding M. A nomenclature for ligand-gated ion channels. Neuropharmacology (2009) 56:2–5. doi: 10.1016/j.neuropharm.2008.06.063 18655795 PMC2847504

[23] Monaghan DT Bridges RJ Cotman CW. The excitatory amino acid receptors: their classes, pharmacology, and distinct properties in the function of the central nervous system. Annu Rev Pharmacol Toxicol (1989) 29:365–402. doi: 10.1146/annurev.pa.29.040189.002053 2543272

[24] Duvoisin RM Zhang C Ramonell K. A novel metabotropic glutamate receptor expressed in the retina and olfactory bulb. J Neurosci (1995) 15:3075–83. doi: 10.1523/JNEUROSCI.15-04-03075.1995 PMC6577763 7722646

[25] Conn PJ Pin JP. Pharmacology and functions of metabotropic glutamate receptors. Annu Rev Pharmacol Toxicol (1997) 37:205–37. doi: 10.1146/annurev.pharmtox.37.1.205 9131252

[26] Ferraguti F Shigemoto R. Metabotropic glutamate receptors. Cell Tissue Res (2006) 326:483–504. doi: 10.1007/s00441-006-0266-5 16847639

[27] Quraishi S Gayet J Morgans CW Duvoisin RM. Distribution of group-III metabotropic glutamate receptors in the retina. J Comp Neurol (2007) 501:931–43. doi: 10.1002/cne.21274 17311335

[28] Nomura A Shigemoto R Nakamura Y Okamoto N Mizuno N Nakanishi S. Developmentally regulated postsynaptic localization of a metabotropic glutamate receptor in rat rod bipolar cells. Cell (1994) 77:361–9. doi: 10.1016/0092-8674(94)90151-1 8181056

[29] Pan F Mills SL Massey SC. Screening of gap junction antagonists on dye coupling in the rabbit retina. Vis Neurosci (2007) 24:609–18. doi: 10.1017/S0952523807070472 PMC2213422 17711600

[30] Li W Keung JW Massey SC. Direct synaptic connections between rods and OFF cone bipolar cells in the rabbit retina. J Comp Neurol (2004) 474:1–12. doi: 10.1002/cne.20075 15156575

[31] Li W Trexler EB Massey SC. Glutamate receptors at rod bipolar ribbon synapses in the rabbit retina. J Comp Neurol (2002) 448:230–48. doi: 10.1002/cne.10189 12115706

[32] Firth SI Li W Massey SC Marshak DW. AMPA receptors mediate acetylcholine release from starburst amacrine cells in the rabbit retina. J Comp Neurol (2003) 466:80–90. doi: 10.1002/cne.10880 14515241 PMC3341736

[33] Schmitz F Konigstorfer A Sudhof TC. RIBEYE, a component of synaptic ribbons: a protein's journey through evolution provides insight into synaptic ribbon function. Neuron (2000) 28:857–72. doi: 10.1016/S0896-6273(00)00159-8 11163272

[34] tom Dieck S Altrock WD Kessels MM Qualmann B Regus H Brauner D. Molecular dissection of the photoreceptor ribbon synapse: physical interaction of Bassoon and RIBEYE is essential for the assembly of the ribbon complex. J Cell Biol (2005) 168:825–36. doi: 10.1083/jcb.200408157 PMC2171818 15728193

[35] DeVries SH Schwartz EA. Kainate receptors mediate synaptic transmission between cones and 'Off' bipolar cells in a mammalian retina. Nature (1999) 397:157–60. doi: 10.1038/16462 9923677

[36] DeVries SH. Bipolar cells use kainate and AMPA receptors to filter visual information into separate channels. Neuron (2000) 28:847–56. doi: 10.1016/S0896-6273(00)00158-6 11163271

[37] Haverkamp S Grünert U Wässle H. Localization of kainate receptors at the cone pedicles of the primate retina. J Comp Neurol (2001) 436:471–86. doi: 10.1002/cne.1081 11447590

[38] Puller C Ivanova E Euler T Haverkamp S Schubert T. OFF bipolar cells express distinct types of dendritic glutamate receptors in the mouse retina. Neuroscience (2013) 243:136–48. doi: 10.1016/j.neuroscience.2013.03.054 23567811

[39] Lindstrom SH Ryan DG Shi J DeVries SH. Kainate receptor subunit diversity underlying response diversity in retinal off bipolar cells. J Physiol (2014) 592:1457–77. doi: 10.1113/jphysiol.2013.265033 PMC3979605 24396054

[40] Puthussery T Percival KA Venkataramani S Gayet-Primo J Grünert U Taylor WR. Kainate receptors mediate synaptic input to transient and sustained OFF visual pathways in primate retina. J Neurosci (2014) 34:7611–21. doi: 10.1523/JNEUROSCI.4855-13.2014 PMC4035522 24872565

[41] Ichinose T Hellmer CB. Differential signalling and glutamate receptor compositions in the OFF bipolar cell types in the mouse retina. J Physiol (2016) 594:883–94. doi: 10.1113/JP271458 PMC4753269 26553530

[42] Ahnelt P Keri C Kolb H. Identification of pedicles of putative blue-sensitive cones in the human retina. J Comp Neurol (1990) 293:39–53. doi: 10.1002/cne.902930104 2312791

[43] Boycott BB Hopkins JM Sperling HG. Cone connections of the horizontal cells of the rhesus monkey's retina. Proc R Soc London Ser B Biol Sci (1987) 229:345–79. doi: 10.1098/rspb.1987.0001 2881306

[44] Wässle H Boycott BB Rohrenbeck J. Horizontal Cells in the Monkey Retina: Cone connections and dendritic network. Eur J Neurosci (1989) 1:421–35. doi: 10.1111/j.1460-9568.1989.tb00350.x 12106129

[45] Boycott BB Kolb H. The horizontal cells of the rhesus monkey retina. J Comp Neurol (1973) 148:115–39. doi: 10.1002/cne.901480107 4121525

[46] Grünert U Martin PR. Cell types and cell circuits in human and non-human primate retina. Prog Retin Eye Res (2020) 78:100844. doi: 10.1016/j.preteyeres.2020.100844 32032773

[47] Pan F Massey SC. Rod and cone input to horizontal cells in the rabbit retina. J Comp Neurol (2007) 500:815–31. doi: 10.1002/cne.21127 17177254

[48] Boycott BB Peichl L Wässle H. Morphological types of horizontal cell in the retina of the domestic cat. Proc R Soc Lond B Biol Sci (1978) 203:229–45. doi: 10.1098/rspb.1978.0103 84387

[49] Ahnelt P Kolb H. Horizontal cells and cone photoreceptors in human retina: a Golgi-electron microscopic study of spectral connectivity. J Comp Neurol (1994) 343:406–27. doi: 10.1002/cne.903430306 8027450

[50] Hack I Peichl L Brandstätter JH. An alternative pathway for rod signals in the rodent retina: rod photoreceptors, cone bipolar cells, and the localization of glutamate receptors. Proc Natl Acad Sci USA (1999) 96:14130–5. doi: 10.1073/pnas.96.24.14130 PMC24202 10570210

[51] Dacheux RF Raviola E. Horizontal cells in the retina of the rabbit. J Neurosci (1982) 2:1486–93. doi: 10.1523/JNEUROSCI.02-10-01486.1982 PMC6564416 6181232

[52] Massey SC O'brien JJ Trexler EB Li W Keung JW Mills SL. Multiple neuronal connexins in the mammalian retina. Cell Communication Adhesion (2003) 10:425–30. doi: 10.1080/cac.10.4-6.425.430 14681052

[53] Hombach S Janssen-Bienhold U Söhl G Schubert T Bussow H Ott T. Functional expression of connexin57 in horizontal cells of the mouse retina. Eur J Neurosci (2004) 19:2633–40. doi: 10.1111/j.0953-816X.2004.03360.x 15147297

[54] Palacios-Prado N Bukauskas FF. Heterotypic gap junction channels as voltage-sensitive valves for intercellular signaling. Proc Natl Acad Sci USA (2009) 106:14855–60. doi: 10.1073/pnas.0901923106 PMC2736430 19706392

[55] O'brien JJ Li W Pan F Keung J O'brien J Massey SC. Coupling between A-type horizontal cells is mediated by connexin 50 gap junctions in the rabbit retina. J Neurosci (2006) 26:11624–36. doi: 10.1523/JNEUROSCI.2296-06.2006 PMC6674794 17093084

[56] Pan F Keung J Kim IB Snuggs MB Mills SL O'brien J. Connexin 57 is expressed by the axon terminal network of B-type horizontal cells in the rabbit retina. J Comp Neurol (2012) 520:2256–74. doi: 10.1002/cne.23060 PMC4568982 22495514

[57] Klaassen LJ Fahrenfort I Kamermans M. Connexin hemichannel mediated ephaptic inhibition in the retina. Brain Res (2012) 1487:25–38. doi: 10.1016/j.brainres.2012.04.059 22796289

[58] Ströh S Sonntag S Janssen-Bienhold U Schultz K Cimiotti K Weiler R. Cell-specific cre recombinase expression allows selective ablation of glutamate receptors from mouse horizontal cells. PloS One (2013) 8:e83076. doi: 10.1371/journal.pone.0083076 24349437 PMC3861464

[59] Grabner CP Futagi D Shi J Bindokas V Kitano K Schwartz EA. Mechanisms of simultaneous linear and nonlinear computations at the mammalian cone photoreceptor synapse. Nat Commun (2023) 14:3486. doi: 10.1038/s41467-023-38943-2 37328451 PMC10276006

[60] Zenisek D Horst NK Merrifield C Sterling P Matthews G. Visualizing synaptic ribbons in the living cell. J Neurosci (2004) 24:9752–9. doi: 10.1523/JNEUROSCI.2886-04.2004 PMC6730242 15525760

[61] Li W DeVries SH. Bipolar cell pathways for color and luminance vision in a dichromatic mammalian retina. Nat Neurosci (2006) 9:669–75. doi: 10.1038/nn1686 16617341

